# Trends in mortality related to kidney failure and diabetes mellitus in the United States: a 1999–2020 analysis

**DOI:** 10.1007/s40620-024-01990-z

**Published:** 2024-06-25

**Authors:** Ahmed Mustafa Rashid, Adeena Jamil, Zoha Khan, Muteia Shakoor, Usama Hussain Kamal, Iqra Israr Khan, Abdullah Akram, Mariam Shahabi, Naser Yamani, Soha Ali, Kanza Fatima, Aamna Kamdi, Muhammad Junaid, Ayesha Mazhar Khan, Jishanth Mattumpuram, Prinka Perswani

**Affiliations:** 1 Department of Medicine, Jinnah Sindh Medical University, Karachi, Pakistan; 2 Department of Medicine, Dow International Medical College, Dow University of Health Sciences, Karachi, Pakistan; 3 Department of Medicine, Services Institute of Medical Sciences, Lahore, Pakistan; 4 Department of Medicine, Dow Medical College, Dow University of Health Sciences, Karachi, Pakistan; 5 Division of Cardiology, Department of Medicine, University of Arizona, Phoenix, AZ USA; 6 Department of Medicine, D.G Khan Medical College, Dera Ghazi Khan, Pakistan; 7 Rashid Latif Medical College, Lahore, Pakistan; 8 Division of Cardiology, Department of Medicine, University of Louisville, Louisville, KY USA; 9 University of California, Riverside, USA

**Keywords:** Diabetes mellitus, Kidney failure, Mortality trends, Racial disparities

## Abstract

**Background:**

Kidney failure ranks as the tenth leading cause of mortality in the United States (US), frequently arising as a complication associated with diabetes mellitus (DM).

**Methods:**

Trends in DM and kidney failure mortality were assessed using a cross-sectional analysis of death certificates from the Centers for Disease Control and Prevention Wide-Ranging Online Data for Epidemiologic Research (CDC WONDER) database. Crude and age-adjusted mortality rates (AAMR) per 100,000 people and annual percent change (APC) in age-adjusted mortality rate with 95% CI were obtained and measured across different demographic and geographic subgroups.

**Results:**

Between 1999 and 2020, a total of 325,515 deaths occurred related to kidney failure and DM. The overall age-adjusted mortality rate showed no significant change between 1999 and 2012, after which it declined until 2015 − 64.8 (95% CI − 75.6 to − 44.8) and has been steadily increasing since. Men had consistently higher age-adjusted mortality rates than women throughout the study duration (overall age-adjusted mortality rate men: 8.1 vs. women: 5.9). Non-Hispanic (NH) Black or African American individuals had the highest overall age-adjusted mortality rate (13.9), followed by non-Hispanic American Indian or Alaskan Native (13.7), Hispanic or Latino (10.3), non-Hispanic Asian or Pacific Islander (6.1), and non-Hispanic White (6.0). Age-adjusted mortality rate also varied by region (overall age-adjusted mortality rate: West:7.5; Midwest: 7.1; South: 6.8; Northeast: 5.8), and non metropolitan areas had higher overall age-adjusted mortality rate (7.5) than small/medium (7.2) and large metropolitan areas (6.4).

**Conclusion:**

After an initial decline, mortality rose across all the demographic groups from 2015 to 2020, revealing notable disparities in gender, race, and region.

**Graphical Abstract:**

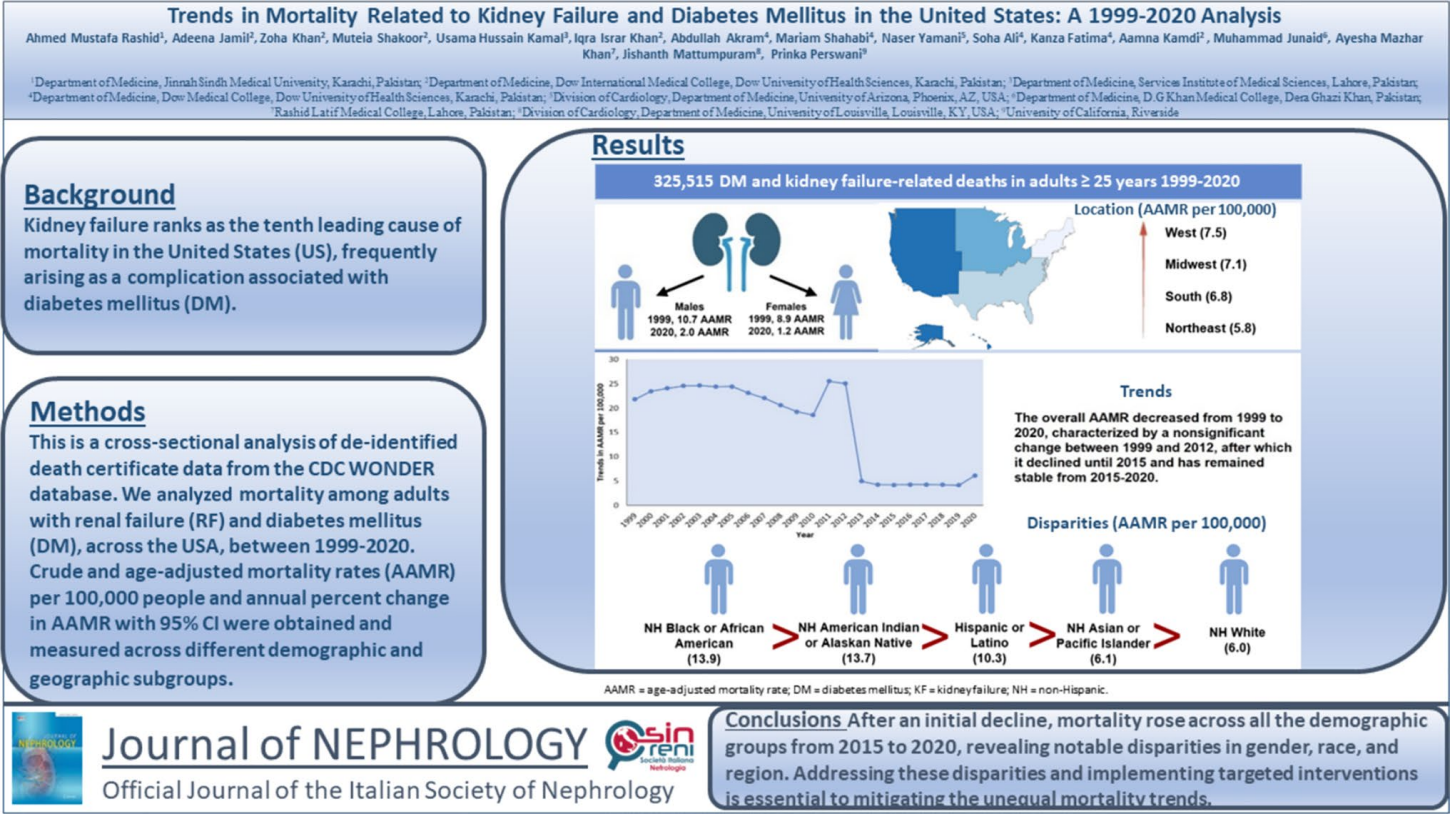

**Supplementary Information:**

The online version contains supplementary material available at 10.1007/s40620-024-01990-z.

## Introduction

Existing Global Burden of Diseases, Injuries, and Risk Factors Study (GBD) results ranked diabetes mellitus (DM) as the eighth leading cause of mortality and morbidity globally [1]. The American Diabetes Association (ADA) has estimated that the disease cost the United States (US) economy over $300 billion in 2017, with nearly 1.4 million Americans being diagnosed with diabetes every year [2, 3]. The association of diabetes with other comorbidities has been widely acknowledged. Rodriguez et al. assessed diabetic mortality in the United States, using a multiple-causes-of-death approach, and found that death certificates that listed DM as an underlying, or primary, cause of death were three times as likely to also mention kidney disease and hypertension on the same [4].

Diabetes mellitus is a primary driver of chronic kidney disease (CKD), the tenth leading cause of mortality in the United States. Chronic kidney disease can progress to end-stage kidney disease (ESKD) or kidney failure [5]. End-stage kidney disease presents a growing healthcare concern, affecting a global population of over 75 million [6], with 4.902 to 7.083 million individuals requiring renal replacement therapy [7]. In 2019, Medicare’s fees for service expenses related to ESKD amounted to 37.3 billion dollars, accounting for 7% of total Medicare costs [8]. This rose to 50.8 billion dollars in 2020 [9]. Given the projection of a 54% increase in diabetes prevalence in the United States by 2030 compared to 2015, a corresponding rise in the prevalence of CKD is anticipated [10].

Therefore, we sought to comprehensively understand the mortality associated with DM and kidney failure. We utilized the nationwide CDC WONDER (Centers for Disease Control and Prevention Wide-Ranging Online Data for Epidemiologic Research) database to examine demographic and regional mortality trends associated with DM and kidney failure from 1999 to 2020.

## Methods

### Data source

The analysis of the mortality trends in adults with kidney failure and DM across the United States of America was performed using the data from the CDC WONDER database [11]. The International Classification of Diseases, Tenth Revision (ICD-10) codes N18 and E10–E14 for kidney failure and DM, respectively, were utilized for the identification of the records. The multiple causes of death public use records and death certificates were examined to discern kidney failure and DM as contributing or underlying causes of death. Approval from the institutional review board was not necessary as only publicly available data were used for the analysis.

### Data extraction

Data were retrieved from the CDC WONDER database for all 51 states in the United States of America, spanning from 1999 to 2020. The data included information regarding demographics, population size, year, and geographical location. Demographic data consists of gender, age, race, and ethnicity. The genders included were male and female, as specified on the death certificates, and were of age ≥ 25 years old. Pre-specified age groups were determined to categorize age into 25–34, 35–44, 45–54, 55–64, 65–74, 75–84, and ≥ 85 years. Race and ethnicity are stated as non-Hispanic (NH) White, non-Hispanic Black or African American, Hispanic or Latino, non-Hispanic American Indian or Alaskan Native, and non-Hispanic Asian or Pacific Islander individuals as per the CDC WONDER database. The geographical data were categorized into urban, large metropolitan areas [population ≥ 1 million], medium/small metropolitan areas [population 50 000–999 999], and rural areas, population < 50 000, as per the National Center for Health Statistics' (NCHS) Urban–Rural Classification Scheme for Counties. Furthermore, the regions were defined as Northeast, Midwest, South, and West [12].

### Statistical analysis

The crude and age-adjusted mortality rates (AAMR) per 100,000 people were used to assess the nationwide trends in kidney failure and DM mortality rates. The crude mortality rate (CMR) was calculated by dividing the total number of kidney failure and DM-related deaths that year by the equivalent U.S. population. Age-adjusted mortality rate was determined by standardizing kidney failure and DM-related deaths to the 2000 population [13]. The annual percent change (APC) in age-adjusted mortality rate with a 95% CI was analyzed using the Joinpoint Regression Program (Joinpoint V 5.0, National Cancer Institute) to recognize the significance of the annual patterns [14]. The significant changes in age-adjusted mortality rate for kidney failure and DM were determined by fitting long linear regression models and identifying the temporal variations. Annual percent change was deemed to be either increasing or decreasing if the slope representing the change in mortality significantly differed from zero. This was determined using two-tailed *t*-tests. A P value of < 0.05 was considered to be statistically significant.

## Results

### Annual trends for kidney failure and diabetes mellitus

**Fig. 1:**
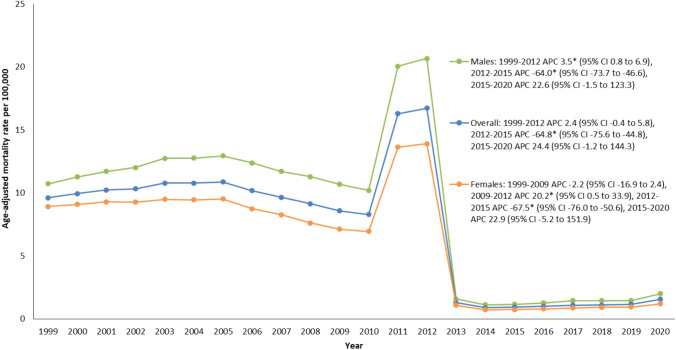
Trends in overall and sex-stratified age-adjusted kidney failure and DM-related mortality rates among adults in the United States, 1999 to 2020. *Indicates that the annual percentage change (APC) significantly differs from zero at α = 0.05

From 1999 to 2020, there were 325,515 reported fatalities associated with kidney failure and diabetes mellitus (Supplemental Table 1). Overall, the age-adjusted mortality rates for DM and kidney failure-related fatalities exhibited a decline from 1999 to 2020, decreasing from 9.6 in 1999 to 1.6 in 2020 (Supplemental Table 2). From 1999 to 2012, there was no significant change in age-adjusted mortality rates. However, from 2012 to 2015, a substantial decrease in age-adjusted mortality rates was observed with an associated annual percent change of − 64.8 (95% CI − 75.6 to − 44.8). Subsequently, from 2015 to 2020, the age-adjusted mortality rates remained relatively stable, maintaining a steady incline (Fig. 1).

### Kidney failure & DM-related age-adjusted mortality rates stratified by gender

Males had persistently elevated age-adjusted mortality rates in comparison to females throughout the duration of the study (overall age-adjusted mortality rates: 8.1 vs 5.9) (Supplemental Table 2). Notably, age-adjusted mortality rates remained rather consistent for males from 1999 to 2012. Whereas females experienced a rise from 2009 to 2012 with an associated annual percent change of 20.2 (95% CI 0.5 to 33.9). However, between 2012 to 2015, both males and females experienced a significant drop in age-adjusted mortality rates with associated annual percent changes of − 64.0 (95% CI − 73.7 to − 46.6) and − 67.5 (95% CI − 76.0 to − 50.6), respectively. Following that, both genders' age-adjusted mortality rates maintained a period of steady incline between 2015 and 2020 (Fig. 1).

### Kidney failure & DM-related age-adjusted mortality rates stratified by race/ethnicity

When stratified by race or ethnicity, the highest age-adjusted mortality rate was observed among non-Hispanic Black or African American individuals (13.9), followed by non-Hispanic American Indian or Alaskan Native (13.7), Hispanic or Latino (10.3), non-Hispanic Asian or Pacific Islander (6.1), and non-Hispanic White (6.0) (Supplemental Table 3).

From 1999 to 2012, age-adjusted mortality rates remained steady across non-Hispanic American Indian or Alaskan Native and non-Hispanic White groups. However, non-Hispanic Black or African Americans, Hispanic or Latinos and non-Hispanic Asian or Pacific Islanders followed a similar trend with consistent age-adjusted mortality rates from 1999 to 2009, followed by a steep rise from 2009 to 2012, with the corresponding annual percent change values of 21.0, 19.6; and 24.4, respectively.

**Fig. 2:**
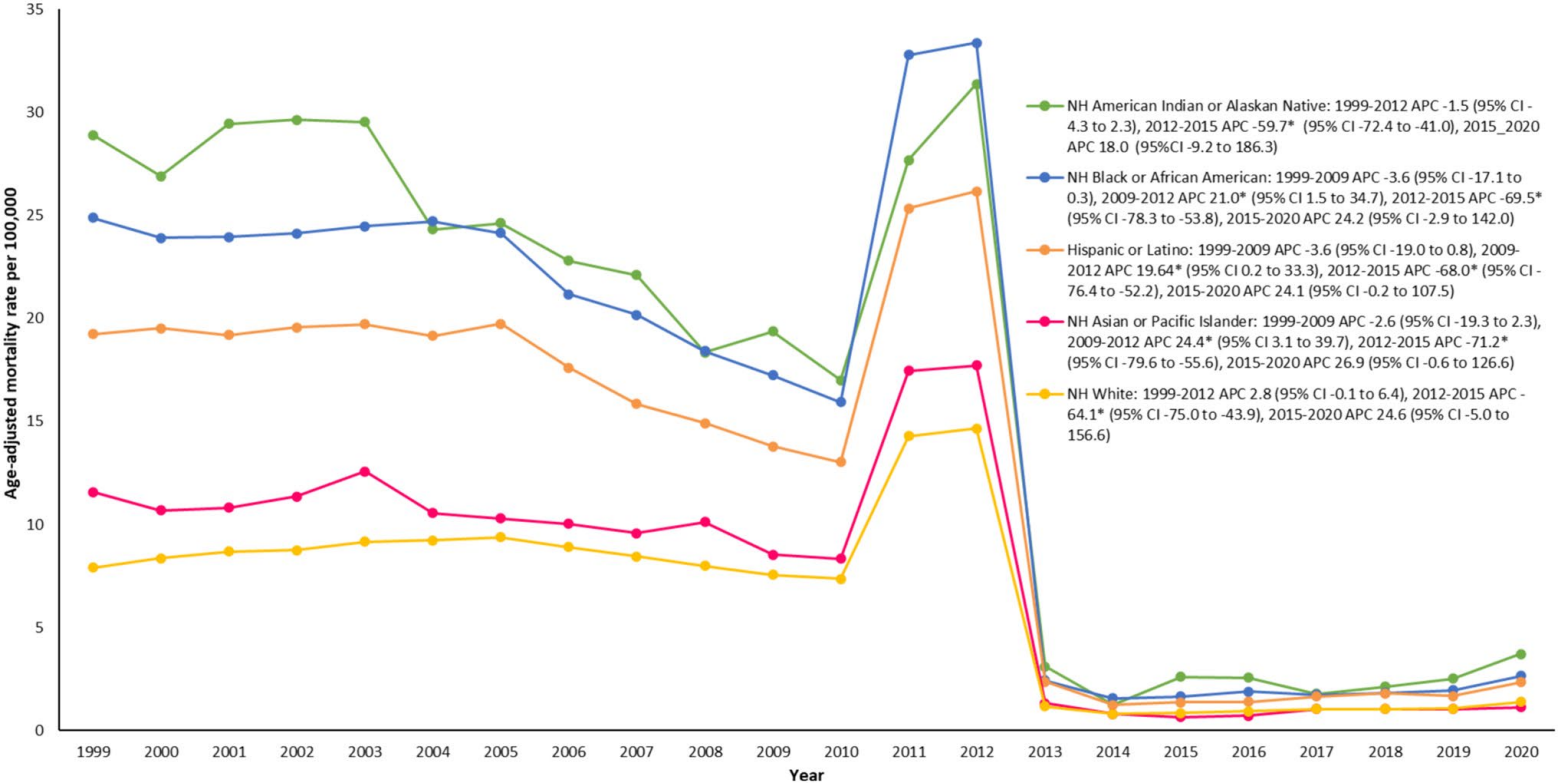
Trends in age-adjusted kidney failure and DM-related mortality rates stratified by race/ethnicity among adults in the United States, 1999 to 2020. *Indicates that the annual percentage change (APC) significantly differs from zero at α = 0.05

During the period from 2012 to 2015, there was a noteworthy and consistent decline in age-adjusted mortality rates across all racial groups, as indicated by annual percent changes: non-Hispanic American Indian or Alaska Native (annual percent change: − 59.7; 95% CI − 72.4, − 41.0). non-Hispanic Black or African American (annual percent change: − 69.5; 95% CI − 78.3, − 53.8), Hispanic or Latino (annual percent change: − 68.0; 95% CI − 76.4, − 52.2), non-Hispanic Asian or Pacific Islander (annual percent change: − 71.2; 95% CI − 79.6, − 55.6), and non-Hispanic White (annual percent change: − 64.1; 95% CI − 75.0, − 43.9). In contrast, from 2015 to 2020, there was a steady upward trend in age-adjusted mortality rates among all racial groups. (Fig. 2).

### Kidney failure & DM-related age-adjusted mortality rates stratified by age

Throughout the study period, there was a consistent upward trend in crude mortality rates with increasing age. Among adults aged over 85 years, crude mortality rates were notably the highest, reaching an overall rate of 13.2. Specifically, in 1999, the crude mortality rate stood at 37.9 before decreasing to 10.0 by 2020 (Supplemental Table 4). Between 2012 and 2015, there was a significant decline in age-adjusted mortality rates across all age groups. However, from 2015 to 2020, the age-adjusted mortality rates for all age groups demonstrated a steady rise. (Supplemental Figs. 1A, B).

### Kidney failure & DM-related age-adjusted mortality rates stratified by geographical region

**Fig. 3:**
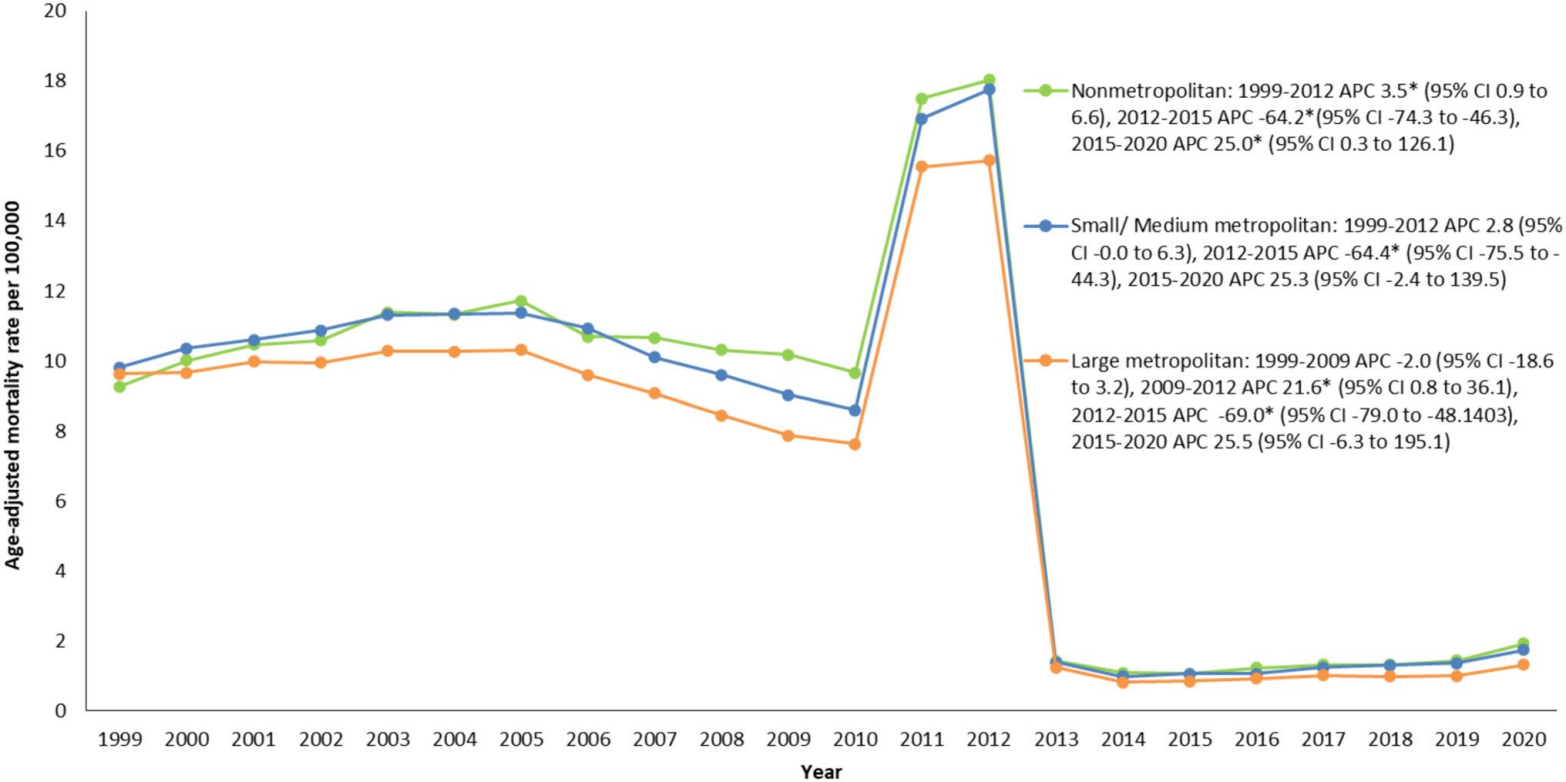
Trends in age-adjusted kidney failure and DM-related mortality rates stratified by rural–urban among adults in the United States, 1999 to 2020. *Indicates the annual percent change (APC) is significantly different than zero at α = 0.05

Throughout our study, significant disparities were observed in mortality rates across diverse regions within the United States. Specifically, the Western region emerged with the highest age-adjusted mortality rate, standing at 7.5, followed closely by the Midwestern region at 7.1. The Southern region demonstrated an age-adjusted mortality rate of 6.8, while the North-eastern region displayed the lowest age-adjusted mortality rate, calculated at 5.8 (Supplemental Table 6, Fig. 3).

Non metropolitan areas consistently reported higher age-adjusted mortality rates (overall age-adjusted mortality rate: 7.5), followed by small/ medium metropolitan areas (overall age-adjusted mortality rate: 7.2), and large metropolitan areas which displayed the lowest age-adjusted mortality rates throughout the study period (overall age-adjusted mortality rate: 6.4). Notable reductions in age-adjusted mortality rates were observed in all areas from 1999 to 2020. Initially, from 1999 to 2012, an increase was seen in age-adjusted mortality rates for non metropolitan (and small/ medium metropolitan) areas, with the increase being significant in non metropolitan areas. Comparatively, a slight decrease in age-adjusted mortality rates was observed for large metropolitan areas from 1999 to 2009. However, a shift in trends occurred during 2011 and 2012, marked by a near two-fold significant increase in age-adjusted mortality rates for large metropolitan areas (2009–2012 annual percent change: 21.6; 95% CI: 0.8 to 36.1). Subsequently, age-adjusted mortality rates across these regions witnessed a unanimous substantial decline from 2012 to 2013 with significant negative annual percent change values during 2012–2015 across non metropolitan (− 64.2; 95% CI: − 74.3 to − 46.3), small/ medium metropolitan (− 64.4; 95% CI: − 75.5 to − 44.3), and large metropolitan areas (− 69.0; 95% CI: − 79.0 to − 48.1403). The age-adjusted mortality rates increased from 2015 to 2020 across all categories with the change being significant in non metropolitan areas (annual percent change: 25.0; 95% CI 0.3 to 126.1) (Supplemental Table 5, Supplemental Fig. 2).

## Discussion

In this study, we report key findings concerning DM and kidney failure-related mortality in the United States over the last two decades. First, following an initial period of decline in mortality rates, a progressive increase in mortality was noted among all demographic groups from 2015 to 2020. Second, males had higher age-adjusted mortality rates throughout the study duration. Third, non-Hispanic Black or African American individuals reported the highest mortality rates in comparison to other racial groups. Fourth, significant geographical disparities emerged, with higher age-adjusted mortality rates in the western region and non metropolitan areas. These observations hold significant implications for public health policy.

Our study revealed a substantial reduction in mortality rates among diverse subgroups of patients diagnosed with both CKD and DM between 1999 and 2020. This decline mirrors similar trends observed in previous studies. For instance, the study conducted by Salichs et al. [15], which exclusively examined patients with CKD, similarly reported a significant decrease in mortality rates across all subgroups during the same timeframe. Furthermore, several other studies [16, 17] focusing on patients with diabetes mellitus specifically observed a parallel downward trajectory in mortality rates among all subgroups over this period. The overall decline in DM and kidney failure-related mortality in the past two decades can be attributed to advancements in healthcare and public awareness with lifestyle modifications. Approximately 60 drugs are currently approved by the Food and Drug Administration (FDA) for the treatment of DM, with the majority approved after 1999 [18]. Web-based programs promoting self-management have proven effective in improving adherence to lifestyle changes in DM and CKD [19]. Notably, advancements such as continuous glucose monitoring and automated insulin delivery systems have improved pre-dialysis glycemic control, reducing mortality [20]. Comprehensive care techniques targeting both the core ailment and associated health conditions have resulted in better management of complications, reducing the overall burden of diabetes and kidney failure in patients [15].

Additionally, our study highlights an increase in mortality observed from 2010 to 2012 which was followed by a substantial reduction in age-adjusted mortality rates across all demographics between 2012 and 2015. During this period, although the incidence was increasing, it has declined due to novel therapies. This supplements the findings of Mokdad et al. who reported an increase in diabetes-related mortality until 2000 with a subsequent decline until 2014, especially at a time when its prevalence was increasing [21]. On the contrary, CKD mortality rates were found to maintain a consistent sharp incline in recent decades in the United States with minimal improvement in prevalence [22]. These contradictory trends in CKD and diabetes mortality might serve to explain our findings. The 2012–2015 decline aligns with the implementation of the 2010 Affordable Care Act (ACA), introducing provisions like expanded insurance coverage and Medicaid expansion, likely facilitating early detection and management of kidney failure [15]. Additionally, the introduction of novel anti-diabetic medications like glucagon-like peptide-1 (GLP-1) agonists and sodium-glucose transport protein 2 (SGLT2) inhibitors, along with advancements in managing chronic conditions like hypertension and DM, may have contributed to improving overall health outcomes during this period [23].

However, we noted a concerning rise in mortality rates related to DM and kidney failure among adults after 2015. This increase can be attributed to factors like the growing prevalence of risk factors like obesity and hypertension. Moreover, despite healthcare advancements, patients with chronic CKD remain susceptible to cardiovascular-related complications, evidencing increased cardiovascular-related mortality rates even in early stages of CKD [24]. As CKD progresses, cardiovascular risk markedly rises. In late-stage CKD (stages 4–5), approximately 50% of patients experience cardiovascular complications. Those with estimated glomerular filtration rate (eGFR) < 60 mL/min per 1.73 m^2^ face double the risk of atrial fibrillation (AF) and acute coronary syndrome. Additionally, heart failure (HF) incidence triples in such patients, and heart failure is closely associated with CKD progression, hospitalization, and mortality. Atrial fibrillation, in turn, triples the risk of progression to ESKD [25]. This underscores the critical need for continued vigilance and comprehensive strategies addressing not only the primary conditions but also associated risk factors while prioritizing early detection and control of blood pressure, diabetes and cardiovascular complications in CKD patients which is crucial to reduce diabetes and CKD burden and mortality.

Our findings also outline significant differences in mortality between males and females. Globally, CKD is more prevalent in women than men [26]. Yet, in this study males demonstrated persistently higher age-adjusted mortality rates associated with DM and kidney failure. The longer life expectancy of females, coupled with the natural decline of kidney function with age, contributes to a larger population at risk of CKD [27]. In contrast, numerous population-based studies demonstrate an alarmingly faster decline in kidney function in men [28, 29]. This inconsistency between prevalence and mortality may stem from gender-related biological variations, leading to distinct GFR decline rates while potential biases in GFR estimation formulas could result in an overestimation of CKD prevalence in women.

Non-Hispanic Black or African American individuals had higher rates of DM and kidney failure-associated mortality throughout our study in comparison to other racial and ethnic groups. The prevalence of kidney failure necessitating renal replacement therapy is fourfold higher among African Americans than in non-Hispanic Whites. African Americans bear the heaviest burden of CKD and diabetes [30, 31]. These disparities may be attributed to genetic factors [32]. Moreover, other factors such as socioeconomic disadvantages and healthcare inequalities may contribute to these differences [32].

Moreover, age emerged as a pivotal determinant in the intricate interplay of factors leading to kidney failure-associated deaths in DM. The increase in crude mortality rate with age is directly associated with the anatomical and physiological changes of the aging kidney and with a longer duration of diabetes [33]. The elevated level of Advanced Glycation End Products (AGEs) and activation of the Receptor for AGEs (RAGE) in senior diabetics cause chronic inflammation and tissue damage, which link to both diabetic kidney disease (DKD) and increased mortality [34].

Our results show that non metropolitan areas consistently reported higher age-adjusted mortality rates in older patients with kidney failure and diabetes mellitus than metropolitan areas. This could be attributed to socioeconomic disparities. Previous studies have shown residents of non metropolitan areas report overall worse physical and mental health compared to their metropolitan counterparts. Additionally, health care and preventative services, as well as emergency and specialty care, are less readily available in said areas. In contrast, risk factors for declining health are observed at much higher rates, including, but not limited to, obesity, smoking, diet, and a lack of the required level of physical activity [35].

Our study's ramifications are potentially important for clinical practice and public health policy. In order to lessen racial and ethnic differences in kidney failure-related mortality, tackling social determinants of health, such as a lack of education, poverty, and access to healthy food is needed. Secondly, age-specific risk assessment and management guidelines, with an emphasis on older people with diabetes, is needed to reduce the risk of mortality linked to kidney failure. Thirdly, healthcare systems should prioritize enhancing the infrastructure in underprivileged areas to handle regional disparities. Public health initiatives aimed at high-risk areas should raise awareness about the importance of diabetes treatment.

Several limitations need to be considered. First, the use of ICD-10 codes and reliance on death certificates raises the possibility of mistaken diagnoses or exclusion of diabetes or kidney failure as cause of death. Second, the database does not contain information on disease severity. Third, the database utilized for this study does not provide detailed information on the specific cause of death, since it provides data on individuals who died with CKD or kidney failure and DM, not from it. Fourth, the database does not offer details on therapies nor contains data on social determinants of health, which may affect access to care and, in turn, affect mortality rates.

In conclusion, after an initial decline, a rise in mortality linked to DM and kidney failure was documented across all demographic groups between 2015 and 2020. Notable disparities were observed in terms of gender, race, and region. Addressing these disparities and implementing targeted interventions is essential to mitigating the unequal mortality trends.

## Supplementary Information

Below is the link to the electronic supplementary material.

Supplementary file1 (DOCX 169 KB)

## Data Availability

The datasets generated and/or analyzed during the current study are available from the corresponding author on reasonable request.
