# Supplementary material for: Trends in mortality related to kidney failure and diabetes mellitus in the United States: a 1999–2020 analysis
Source: J Nephrol. 2024 Jun 25;37(7):1833–41. doi: 10.1007/s40620-024-01990-z (PMC11519297; doi:10.1007/s40620-024-01990-z)
Supplement: Supplementary file 1 — Supplementary file1 (DOCX 169 KB) [file 40620_2024_1990_MOESM1_ESM.docx]

**Supplemental Table 1.** Absolute number of kidney failure and DM-related deaths stratified by sex and race in the United States, 1999-2020.

| **Year** | **Overall** | **Females** | **Males** | **NH White** | **NH Black or African American** | | **NH Asian or Pacific Islander** | **NH American Indian or Alaskan Native** | **Hispanic or Latino** |
| --- | --- | --- | --- | --- | --- | --- | --- | --- | --- |
| 1999 | 17038 | 8964 | 8074 | 12292 | | 3991 | 493 | 262 | 1869 |
| 2000 | 17830 | 9295 | 8535 | 13194 | | 3863 | 492 | 281 | 1962 |
| 2001 | 18681 | 9673 | 9008 | 13882 | | 3965 | 531 | 303 | 2028 |
| 2002 | 19155 | 9798 | 9357 | 14187 | | 4041 | 595 | 332 | 2164 |
| 2003 | 20322 | 10211 | 10111 | 15072 | | 4208 | 690 | 352 | 2303 |
| 2004 | 20617 | 10302 | 10315 | 15410 | | 4309 | 607 | 291 | 2336 |
| 2005 | 21194 | 10571 | 10623 | 15929 | | 4333 | 634 | 298 | 2523 |
| 2006 | 20208 | 9851 | 10357 | 15339 | | 3905 | 659 | 305 | 2363 |
| 2007 | 19545 | 9525 | 10020 | 14818 | | 3778 | 655 | 294 | 2220 |
| 2008 | 18831 | 8927 | 9904 | 14281 | | 3553 | 725 | 272 | 2228 |
| 2009 | 18142 | 8546 | 9596 | 13745 | | 3448 | 649 | 300 | 2166 |
| 2010 | 17712 | 8370 | 9342 | 13538 | | 3234 | 657 | 283 | 2139 |
| 2011 | 35700 | 16991 | 18709 | 26948 | | 6855 | 1441 | 456 | 4325 |
| 2012 | 37600 | 17798 | 19802 | 28308 | | 7186 | 1567 | 539 | 4666 |
| 2013 | 3013 | 1439 | 1574 | 2293 | | 535 | 130 | 55 | 443 |
| 2014 | 2115 | 962 | 1153 | 1641 | | 368 | 82 | 24 | 250 |
| 2015 | 2271 | 1041 | 1230 | 1764 | | 388 | 69 | 50 | 293 |
| 2016 | 2522 | 1137 | 1385 | 1931 | | 452 | 86 | 53 | 311 |
| 2017 | 2812 | 1217 | 1595 | 2214 | | 436 | 125 | 37 | 385 |
| 2018 | 2948 | 1313 | 1635 | 2280 | | 485 | 135 | 48 | 436 |
| 2019 | 3096 | 1408 | 1688 | 2381 | | 516 | 141 | 58 | 422 |
| 2020 | 4163 | 1804 | 2359 | 3174 | | 731 | 163 | 95 | 612 |
| Total | 325515 | 159143 | 166372 | 244621 | | 64580 | 11326 | 4988 | 38444 |

NH: Non-Hispanic

**Supplemental Table 2.** Overall and sex-stratified kidney failure and DM-related age-adjusted mortality rates per 100,000 among adults in the United States, 1999 to 2020.

| **Year** | **Overall** | **Male** | **Female** |
| --- | --- | --- | --- |
| 1999 | 9.6 | 10.7 | 8.9 |
| 2000 | 9.9 | 11.3 | 9.1 |
| 2001 | 10.2 | 11.7 | 9.3 |
| 2002 | 10.3 | 12.0 | 9.3 |
| 2003 | 10.8 | 12.7 | 9.5 |
| 2004 | 10.8 | 12.8 | 9.5 |
| 2005 | 10.9 | 13.0 | 9.5 |
| 2006 | 10.2 | 12.4 | 8.8 |
| 2007 | 9.7 | 11.7 | 8.3 |
| 2008 | 9.2 | 11.3 | 7.6 |
| 2009 | 8.6 | 10.7 | 7.1 |
| 2010 | 8.3 | 10.2 | 6.9 |
| 2011 | 16.3 | 20.1 | 13.6 |
| 2012 | 16.7 | 20.7 | 13.9 |
| 2013 | 1.3 | 1.6 | 1.1 |
| 2014 | 0.9 | 1.1 | 0.7 |
| 2015 | 0.9 | 1.2 | 0.8 |
| 2016 | 1.0 | 1.3 | 0.8 |
| 2017 | 1.1 | 1.5 | 0.9 |
| 2018 | 1.1 | 1.5 | 0.9 |
| 2019 | 1.2 | 1.5 | 0.9 |
| 2020 | 1.6 | 2.0 | 1.2 |
| Total | 6.8 | 8.1 | 5.9 |

**Supplemental Table 3.** Kidney failure and DM-related age-adjusted mortality rates per 100,000 among adults stratified by race/ethnicity in the United States, 1999 to 2020.

| **Year** | **NH White** | **NH Black or African American** | **NH Asian or Pacific Islander** | **NH American Indian or Alaskan Native** | **Hispanic or Latino** |
| --- | --- | --- | --- | --- | --- |
| 1999 | 7.9 | 24.9 | 11.6 | 28.9 | 19.2 |
| 2000 | 8.4 | 23.9 | 10.7 | 26.9 | 19.5 |
| 2001 | 8.7 | 24.0 | 10.8 | 29.5 | 19.2 |
| 2002 | 8.8 | 24.1 | 11.4 | 29.6 | 19.6 |
| 2003 | 9.2 | 24.5 | 12.6 | 29.5 | 19.7 |
| 2004 | 9.2 | 24.7 | 10.6 | 24.3 | 19.2 |
| 2005 | 9.4 | 24.1 | 10.3 | 24.6 | 19.7 |
| 2006 | 8.9 | 21.2 | 10.0 | 22.8 | 17.6 |
| 2007 | 8.4 | 20.2 | 9.6 | 22.1 | 15.9 |
| 2008 | 8.0 | 18.4 | 10.1 | 18.3 | 14.9 |
| 2009 | 7.6 | 17.2 | 8.5 | 19.4 | 13.8 |
| 2010 | 7.4 | 16.0 | 8.4 | 17.0 | 13.0 |
| 2011 | 14.3 | 32.8 | 17.5 | 27.7 | 25.3 |
| 2012 | 14.7 | 33.4 | 17.7 | 31.4 | 26.2 |
| 2013 | 1.2 | 2.4 | 1.3 | 3.1 | 2.4 |
| 2014 | 0.8 | 1.6 | 0.8 | 1.3 | 1.3 |
| 2015 | 0.9 | 1.7 | 0.7 | 2.6 | 1.4 |
| 2016 | 0.9 | 1.9 | 0.7 | 2.6 | 1.4 |
| 2017 | 1.0 | 1.8 | 1.1 | 1.8 | 1.7 |
| 2018 | 1.1 | 1.8 | 1.0 | 2.1 | 1.8 |
| 2019 | 1.1 | 2.0 | 1.0 | 2.5 | 1.7 |
| 2020 | 1.4 | 2.7 | 1.1 | 3.7 | 2.4 |
| Total | 6.0 | 13.9 | 6.1 | 13.7 | 10.3 |

NH: Non-Hispanic

**Supplemental Table 4.** Kidney failure and DM-related crude mortality rates per 100,000 among adults stratified by 10-year age groups in the United States, 1999-2020.

| **Year** | **Age 25-34** | **Age 35-44** | **Age 45-54** | **Age 55-64** | **Age 65-74** | **Age 75-84** | **Age 85+** |
| --- | --- | --- | --- | --- | --- | --- | --- |
| 1999 | 0.5 | 1.3 | 4.4 | 13.5 | 28.0 | 38.5 | 37.9 |
| 2000 | 0.4 | 1.2 | 4.3 | 12.8 | 29.1 | 41.3 | 45.7 |
| 2001 | 0.4 | 1.2 | 4.2 | 12.6 | 29.9 | 43.6 | 49.6 |
| 2002 | 0.4 | 1.3 | 3.9 | 12.3 | 29.7 | 45.2 | 54.3 |
| 2003 | 0.4 | 1.3 | 3.8 | 12.5 | 30.6 | 48.8 | 58.6 |
| 2004 | 0.3 | 1.3 | 3.8 | 12.0 | 29.7 | 50.2 | 61.5 |
| 2005 | 0.3 | 1.3 | 3.7 | 11.8 | 28.8 | 51.2 | 68.0 |
| 2006 | 0.4 | 1.2 | 3.4 | 10.8 | 27.1 | 48.0 | 64.9 |
| 2007 | 0.3 | 1.0 | 3.2 | 9.7 | 24.6 | 46.7 | 67.4 |
| 2008 | 0.3 | 1.1 | 2.9 | 9.4 | 23.4 | 43.7 | 63.3 |
| 2009 | 0.3 | 1.0 | 2.9 | 8.8 | 21.7 | 41.2 | 59.8 |
| 2010 | 0.3 | 1.0 | 2.9 | 8.5 | 20.2 | 40.0 | 58.3 |
| 2011 | 0.5 | 1.6 | 5.1 | 14.7 | 38.3 | 81.5 | 132.2 |
| 2012 | 0.4 | 1.7 | 4.8 | 14.9 | 38.4 | 84.1 | 144.0 |
| 2013 | 0.1 | 0.1 | 0.4 | 1.2 | 3.0 | 7.0 | 10.2 |
| 2014 | Suppressed | 0.1 | 0.3 | 0.8 | 2.2 | 4.6 | 6.8 |
| 2015 | Unreliable | 0.1 | 0.3 | 0.8 | 2.2 | 4.9 | 7.6 |
| 2016 | Unreliable | 0.1 | 0.3 | 1.0 | 2.3 | 5.1 | 8.5 |
| 2017 | Unreliable | 0.1 | 0.3 | 1.0 | 2.5 | 5.9 | 8.9 |
| 2018 | 0.1 | 0.1 | 0.3 | 1.0 | 2.6 | 5.6 | 9.8 |
| 2019 | Unreliable | 0.1 | 0.4 | 1.0 | 2.6 | 6.0 | 10.0 |
| 2020 | Unreliable | 0.2 | 0.5 | 1.5 | 3.5 | 7.6 | 13.2 |

**Supplemental Table 5**. Kidney failure and diabetes related age-adjusted mortality rates per 100,000 among adults in the United States, 1999-2020.

| **Census Region** | **Year** | **Age Adjusted Rate** |
| --- | --- | --- |
| Northeast | 1999 | 8.5 |
| Northeast | 2000 | 9.2 |
| Northeast | 2001 | 9.0 |
| Northeast | 2002 | 9.1 |
| Northeast | 2003 | 9.3 |
| Northeast | 2004 | 9.0 |
| Northeast | 2005 | 9.1 |
| Northeast | 2006 | 8.4 |
| Northeast | 2007 | 8.0 |
| Northeast | 2008 | 7.3 |
| Northeast | 2009 | 6.8 |
| Northeast | 2010 | 6.7 |
| Northeast | 2011 | 12.7 |
| Northeast | 2012 | 13.0 |
| Northeast | 2013 | 1.1 |
| Northeast | 2014 | 0.7 |
| Northeast | 2015 | 0.6 |
| Northeast | 2016 | 0.7 |
| Northeast | 2017 | 0.8 |
| Northeast | 2018 | 0.9 |
| Northeast | 2019 | 0.9 |
| Northeast | 2020 | 1.2 |
| Northeast | Total | 5.8 |
| Midwest | 1999 | 9.6 |
| Midwest | 2000 | 9.8 |
| Midwest | 2001 | 10.3 |
| Midwest | 2002 | 10.5 |
| Midwest | 2003 | 10.7 |
| Midwest | 2004 | 11.2 |
| Midwest | 2005 | 11.0 |
| Midwest | 2006 | 10.9 |
| Midwest | 2007 | 10.2 |
| Midwest | 2008 | 9.7 |
| Midwest | 2009 | 9.2 |
| Midwest | 2010 | 8.7 |
| Midwest | 2011 | 16.9 |
| Midwest | 2012 | 17.1 |
| Midwest | 2013 | 1.2 |
| Midwest | 2014 | 0.9 |
| Midwest | 2015 | 1.0 |
| Midwest | 2016 | 1.0 |
| Midwest | 2017 | 1.2 |
| Midwest | 2018 | 1.1 |
| Midwest | 2019 | 1.2 |
| Midwest | 2020 | 1.7 |
| Midwest | Total | 7.1 |
| South | 1999 | 9.9 |
| South | 2000 | 10.4 |
| South | 2001 | 10.7 |
| South | 2002 | 10.6 |
| South | 2003 | 11.1 |
| South | 2004 | 11.0 |
| South | 2005 | 11.2 |
| South | 2006 | 10.1 |
| South | 2007 | 9.6 |
| South | 2008 | 8.9 |
| South | 2009 | 8.7 |
| South | 2010 | 8.2 |
| South | 2011 | 16.1 |
| South | 2012 | 16.4 |
| South | 2013 | 1.3 |
| South | 2014 | 0.9 |
| South | 2015 | 0.9 |
| South | 2016 | 1.0 |
| South | 2017 | 1.0 |
| South | 2018 | 1.1 |
| South | 2019 | 1.1 |
| South | 2020 | 1.5 |
| South | Total | 6.8 |
| West | 1999 | 10.2 |
| West | 2000 | 10.1 |
| West | 2001 | 10.7 |
| West | 2002 | 11.0 |
| West | 2003 | 11.8 |
| West | 2004 | 11.5 |
| West | 2005 | 11.9 |
| West | 2006 | 11.2 |
| West | 2007 | 10.7 |
| West | 2008 | 10.5 |
| West | 2009 | 9.5 |
| West | 2010 | 9.4 |
| West | 2011 | 19.3 |
| West | 2012 | 20.3 |
| West | 2013 | 1.6 |
| West | 2014 | 1.0 |
| West | 2015 | 1.2 |
| West | 2016 | 1.3 |
| West | 2017 | 1.4 |
| West | 2018 | 1.4 |
| West | 2019 | 1.4 |
| West | 2020 | 1.8 |
| West | Total | 7.5 |

**Supplemental Table 6.** Kidney failure and DM-related age adjusted mortality rates per 100,000 among adults stratified by urban-rural classification in the United States, 1999 to 2020.

| **Year** | **Nonmetropolitan** | **Small/ Medium Metropolitan** | **Large Metropolitan** |
| --- | --- | --- | --- |
| 1999 | 9.3 | 9.8 | 9.6 |
| 2000 | 10.0 | 10.4 | 9.7 |
| 2001 | 10.5 | 10.6 | 10.0 |
| 2002 | 10.6 | 10.9 | 9.9 |
| 2003 | 11.4 | 11.3 | 10.3 |
| 2004 | 11.3 | 11.3 | 10.3 |
| 2005 | 11.7 | 11.4 | 10.3 |
| 2006 | 10.7 | 10.9 | 9.6 |
| 2007 | 10.7 | 10.1 | 9.1 |
| 2008 | 10.3 | 9.6 | 8.5 |
| 2009 | 10.2 | 9.0 | 7.9 |
| 2010 | 9.7 | 8.6 | 7.6 |
| 2011 | 17.5 | 16.9 | 15.5 |
| 2012 | 18.0 | 17.8 | 15.7 |
| 2013 | 1.4 | 1.4 | 1.2 |
| 2014 | 1.1 | 1.0 | 0.8 |
| 2015 | 1.1 | 1.1 | 0.8 |
| 2016 | 1.2 | 1.1 | 0.9 |
| 2017 | 1.3 | 1.2 | 1.0 |
| 2018 | 1.3 | 1.3 | 1.0 |
| 2019 | 1.4 | 1.4 | 1.0 |
| 2020 | 1.9 | 1.7 | 1.3 |
| Total | 7.5 | 7.2 | 6.4 |

**Supplementary Figure 1A**. Trends in kidney failure and DM-related crude mortality rates stratified by age groups 35-64 among adults in the United States, 1999 to 2020.

*Indicates the annual percent change (APC) is significantly different than zero at α=0.05

**Supplementary Figure 1B**. Trends in kidney failure and DM-related crude mortality rates stratified by age groups 65 and above among adults in the United States, 1999 to 2020.

*Indicates the annual percent change (APC) is significantly different than zero at α=0.05.

**Supplementary Figure 2.** Kidney failure and DM-related age‐adjusted mortality rates stratified by region among adults in the United States, 1999 to 2020.

**
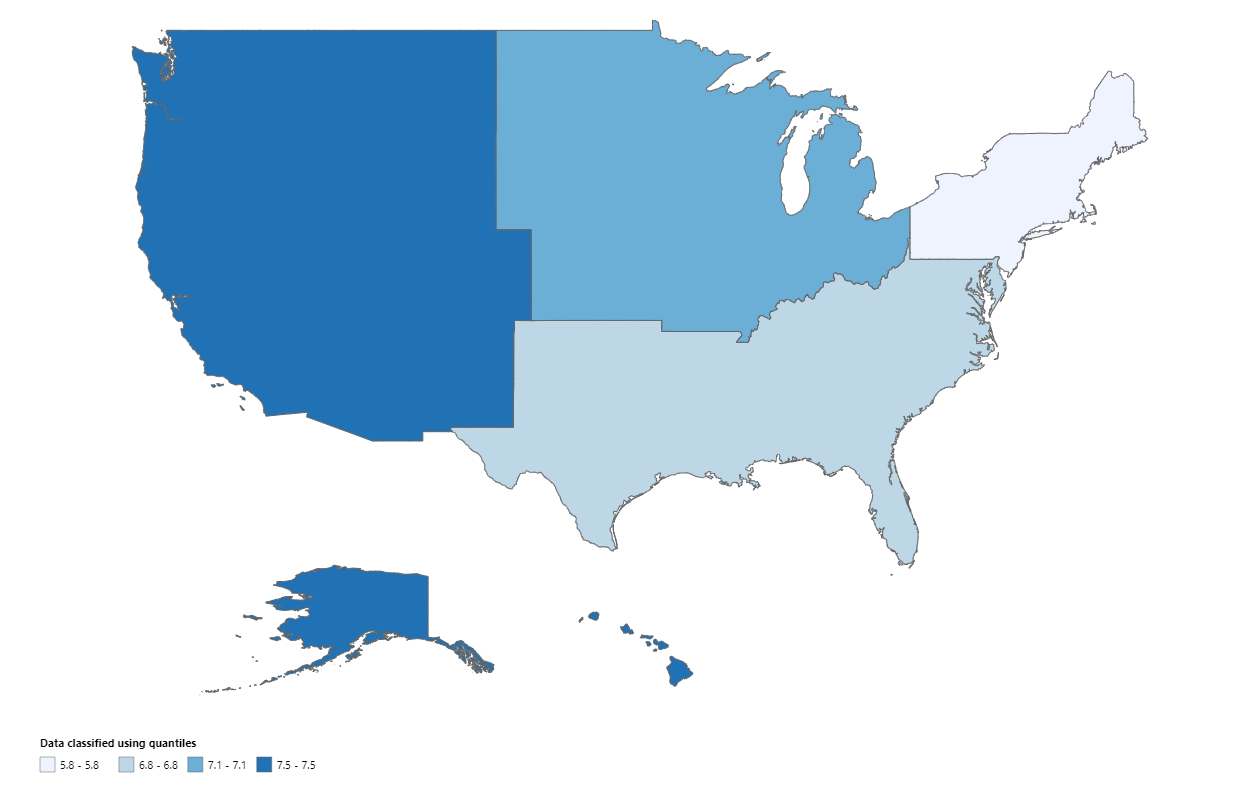
**

**References (continued)**

1. Lea JP, Nicholas SB. Diabetes mellitus and hypertension: key risk factors for kidney disease. J Natl Med Assoc. 2002 Aug;94(8 Suppl):7S-15S. PMID: 12152917; PMCID: PMC2594170.
2. Kovesdy CP. CKD in African Americans as a Complex Intertwining of Biology and Socioeconomics: An Introduction. Am J Kidney Dis. 2018 Nov;72(5 Suppl 1):S1-S2. doi: 10.1053/j.ajkd.2018.06.019. PMID: 30343716.
3. Glassock RJ, Rule AD. The implications of anatomical and functional changes of the aging kidney: with an emphasis on the glomeruli. Kidney Int. 2012;82(3):270-7.
4. Wu XQ, Zhang DD, Wang YN, Tan YQ, Yu XY, Zhao YY. AGE/RAGE in diabetic kidney disease and ageing kidney. Free Radic Biol Med. 2021;171:260-71.
5. Moy E, Garcia MC, Bastian B, Rossen LM, Ingram DD, Faul M, et al. Leading causes of death in nonmetropolitan and metropolitan areas - United States, 1999-2014. MMWR Surveillance Summaries. 2017;66(1).
